# The Great Boat Race

**Published:** 1869-10

**Authors:** 


### THE GREAT BOAT RACE.

Great in one thing, its folly, as are all competitive outdoor games and strifes as practised, such as base-ball, feats acrobatic, tall walking, heavy lifting, gymnastics and the whole brood of time-wasters and destroyers of health and true enjoyment. What good does it do if a man rows a boat a mile in a minute? It does not earn an honest penny; besides nobody has any business to go a mile in a minute. What's the use of young men starving themselves half to death, pinching themselves into thinness of flesh for want of necessary food, and into actual disease? What good can it do a man to inure himself to a practice or employment which he does not expect to follow, and which brings him neither money, health, nor comfort, for there is no comfort in being half starved?

What is the result of all this tom-foolery of walking long distances in consecutive hours? It was never intended for men to work consecutively, or to work at all at night. Then follows walking backwards; next will come efforts to walk upside down in the water, under the water, on the water. Many a man has been put in his grave by cutting monkey capers in a gymnasium, and many others made lame for life. Suppose a man can climb a greased pole fifty feet high, has he any more agility than he had before? There is nothing at the top of a greased pole; besides there is no use for greased poles, and it's a clear waste of soap fat. Some man down East has been making a fool of himself by endeavoring to get the ability, in the course of years, to lift an elephant. Elephants were not intended to be lifted; they can better hoist themselves if there is need for it. If a man wants to climb or lift his own weight, let him stick on a tail, and go it like a monkey; the monkey is wise in comparison with such, for he makes a living at times by climbing trees for nuts or other provender.

Then there is base-ball. Column after column is wasted in our newspapers in details of the innings and winnings of Tom, Dick and Harry, of whom nobody ever heard or cares a button. There is no material difference between a horse-race and a man-race. A feat of fisticuffs and a game of base ball are radically alike in their social, physical, moral and hygienical effects; they all promote idleness, gambling and drunkenness, just as the theatre does. Idlers, gamblers, drunkards, the profane and the obscene visit alike the theatre and the race field, always on hand at a professional "set to," ready for a bet, a knock down, a game of cards, or a drink, multiplied by forty in a day. We hear a great deal of twaddle about the games of the Greeks and Romans, and associate their great deeds of war and conquest with their feats in the amphitheatre. But there is this little drawback; as soon as they got time to run races, they ran to seed; they got drunk; became extravagant; spent more than they earned, sought to make up the deficiency by gambling, and, failing there, abandoned themselves to all kinds of dissipation and recklessness, and then, when in the course of years they became in a sense emasculated, deprived of their manly power of doing and daring great deeds, they set their bulls to fighting one another, and then the bulls to fighting broken-down horses, as the Spaniards now do; and finally, both Greek and Roman died out. Athletes do not live long. The heroes of the ring die early. The "training" which they have to undergo seems to undermine their constitutions. The fact is, all strainings of the human body beyond what it is intended for as a regular pursuit, are injurious to the constitution, are premature drafts on its power, and always will work harm. Man was made for steady labor, for deliberate employment, and those, and those only, promote health and thrift and manly endurance. Look at the time wasted in preparing for competitive games; in actually playing them; in talking about them after they are over; to say nothing about the actual expenditure of money: besides these there are bettings and angry debates, with any number of disagreeable reminiscences. But there is a common source of danger to health in connection with all out-door competitions, aside from the danger of over-training and the various moral evils which have been enumerated, and that is of taking cold after the contests are over. For competitions always leave the body over-heated and wearied, and the mind depressed; the circulation is feeble and languid, and the power to resist cold is very limited indeed; so that a very slight wind can so rapidly convey away the vital heat, that a chill is possible within an hour, to be followed by pleurisy, inflammation of the lungs, or some tedious attack of rheumatism. As soon as any competitive game is over, the parties engaged should go to a warm room and get into bed, and remain there covered until fully rested. The general course, however, is to stand about on the ground and—drink whisky to keep off the cold.

If men want rest from their ordinary occupations and wish to gain strength for further efforts, the first best thing is to go to sleep, then take a joyous walk with some jovial friend, or go a courting, or to the singing-school, or pay a visit to some dear friend or relative, or in some other form of employment as different as possible from the regular occupation, something that is jolly, that has in it the uproarous laugh, to wake up a new circulation and bring into exercise hidden activities.

